# Testing Genomic Offset With Common Gardens in Genetically Structured Black Spruce (*Picea mariana*)

**DOI:** 10.1111/eva.70336

**Published:** 2026-11-14

**Authors:** Anna Fijarczyk, Etienne Robert, Benjamin Marquis, Manuel Lamothe, Patrick Lenz, Martin P. Girardin, Nathalie Isabel

**Affiliations:** ^1^ Natural Resources Canada, Canadian Forest Service, Laurentian Forestry Centre Québec Québec Canada; ^2^ Institut des sciences de la forêt tempérée, Université du Québec en Outaouais Ripon Québec Canada; ^3^ Institut des sciences de l'environnement, Université du Québec à Montréal Montréal Québec Canada; ^4^ Natural Resources Canada, Canadian Forest Service, Great Lakes Forestry Centre Sault Ste. Marie Ontario Canada

**Keywords:** adaptive genetic variation, assisted migration, boreal forests, climate change, Gradient Forest

## Abstract

Boreal forests play a crucial role in regulating climate via storage and release of carbon. Anticipated changes in climate are expected to increase mortality and reduce biomass in many boreal tree species, putting at risk the functioning of these ecosystems and hence their role in carbon sequestration. Genomic offset methods leverage spatial distribution of genomic diversity and its association with environmental variables to predict population vulnerability to projected changes in climate. Here, we analyse over 60 populations and more than 1400 individuals of black spruce ( *Picea mariana* (Mill.) B.S.P), a dominant boreal forest species, to compare population‐level genomic offsets calculated using Gradient Forest and redundancy analysis (RDA) against multiple fitness‐related traits measured across four long‐term (> 40 years) common gardens. Within common gardens, we found that genomic offset projections were largely unaffected by the model choice, the number or type of markers used for model training, with the strongest discrepancies observed for LFMM climate‐associated markers. Model performance remained relatively stable when the number of populations or individuals in the training data set was reduced, suggesting that these models can reliably project genomic offsets for new populations. However, model performances varied among common gardens, with highly accurate fitness projections in some gardens but weak or even contradictory relationships in others. Model performance was influenced by the choice of climate predictors, their relationships with fitness‐related traits, and the genetic cluster in which models were evaluated. Overall, our results highlight the challenges of projecting genomic offsets across large spatial scales in genetically structured species, due to spatial variation in environmental drivers of adaptation and complex interactions among them. By capitalizing on our comprehensive validation, we identified the most robust models for projecting fitness declines in black spruce under future climate scenarios.

## 1 Introduction

Boreal forests comprise 27% of the total forested area on Earth, covering more than 1 billion hectares across the Northern Hemisphere (GlobalFRA, n.d.). They are dominated by conifer species, including spruces (*Picea* spp.) and pines (*Pinus* spp.), that are adapted to short‐growing seasons and cold temperatures. As one of the world's largest terrestrial carbon reservoirs, these boreal forests play an important role in the regulation of global climate by storing and releasing carbon. Boreal forests have been under constant influence of natural disturbances such as extreme climate events, wildfires, insect and disease outbreaks since the retreat of the glaciers about 7000 years ago (Brandt et al. 2013). With projected increase in global temperatures (IPCC 2023), many boreal regions are facing intensification of some of those challenges and increase of drought‐related episodes in some parts of their distribution (Price et al. 2013), putting at risk long‐term functioning of these ecosystems (Gauthier et al. 2015). It is becoming increasingly urgent to understand how boreal tree species will respond to these challenges, and to evaluate the potential benefits of assisted migration for species and populations at risk of maladaptation.

Ecological methods used to predict forest species dynamics under changing environments either model species distributions by examining shifts in habitat suitability across the landscape (Prasad et al. 2020) or model productivity and biomass losses using species‐specific functional traits, such as tree mortality caused by drought or migration failure (Aubin et al. 2018; Liu et al. 2023). However, these approaches do not consider the evolutionary processes shaping intraspecific genetic variation across the landscape, which can lead to divergent populations' responses to the same climate variables. Standing genetic variation within populations has been shaped by past demographic events, such as local extinctions or expansions, by local selective pressures as well as by interactions with other species. Together, these factors ultimately determine the ability of populations to respond to changing environments and can enhance the predictive accuracy of species distribution models (Urban et al. 2016; Aguirre‐Liguori et al. 2021). Conifer tree species often display local adaptation to environmental conditions, as demonstrated with common garden experiments, where population‐level functional traits often vary when growing conditions differ from those found at the seed source (Oleksyn et al. 1998; Housset et al. 2018; Guo et al. 2022; Lortie and Hierro 2022; Messier et al. 2025). Genomic studies further support this by revealing allele frequency changes in genes associated with climate‐related traits (Bergmann 1978; Evans et al. 2014; Depardieu et al. 2021). An increasing number of studies consider population‐level variation in phenotypes or genotypes to model species productivity predictions (St Clair and Howe 2007; Isaac‐Renton et al. 2014; Patsiou et al. 2020; Robert et al. 2024).

Another group of predictive methods aims to estimate genotype–environment mismatch (genomic offset) under climate change using species‐wide genomic variation. Approaches such as Gradient Forest (Ellis et al. 2012; Fitzpatrick and Keller 2015), Risk of Non‐Adaptedness (Rellstab et al. 2016), Latent Factor Mixed Models (Gain and François 2021), or Redundancy Analysis (Capblancq and Forester 2021) model linear or nonlinear relationships between genetic variants and environmental variables across the landscape. These models are then used to project genomic offset under future climate scenarios. Genomic offset is typically calculated as the Euclidean distance between the genomic compositions predicted under current and future climatic conditions. Theoretically, this metric has been linked to changes in fitness when a polygenic trait is under stabilizing selection toward an optimal phenotype (Gain et al. 2023; Capblancq et al. 2025). However, these methods come with their own limitations; for instance, the underlying assumption is that genotypes are most fit in their local climate and that maladaptation to new conditions is instantaneous. Therefore, even though these methods hold promise for straightforward and phenotype‐free risk assessment of species to changing climate (Rellstab et al. 2021), validation studies are needed to assess their utility for conservation or management purposes in different contexts.

To validate genomic offset methods, several studies have conducted either simulations (Láruson et al. 2022; Gain et al. 2023; Lind and Lotterhos 2025a, 2025b) or empirical validation in common gardens in which anticipated climate change in some future period is substituted by different climate conditions experienced by the genotype transplanted to a common garden experiment (Rhoné et al. 2020; Fitzpatrick et al. 2021; Capblancq and Forester 2021; Gain et al. 2023; Lachmuth et al. 2024; Lind et al. 2024; Archambeau et al. 2025; Verrico et al. 2025). The consensus across these studies is that genomic offset is often correlated with fitness and often predicts fitness better than climate or geographical distance alone. However, different models can yield inconsistent patterns of maladaptation (Lind et al. 2024) and the strength of these correlations varies depending on the studied species, as well as data‐ and common garden‐specific factors (Fitzpatrick et al. 2025). Simulation studies demonstrated that the accuracy of genomic offset improves when sampling is denser along environmental gradients (Láruson et al. 2022), and when selection is strong (Lind and Lotterhos 2025b). On the other hand, differences in effective population sizes (Láruson et al. 2022), the presence of novel environments, and the inclusion of noncausal environmental variables can all reduce the accuracy of genomic offset projections (Lind and Lotterhos 2025b). Finally, the choice of markers, whether random, neutral, or adaptive, has been shown to have either a limited impact on genomic offset estimates (Fitzpatrick et al. 2021; Láruson et al. 2022; Lachmuth et al. 2023; Lind et al. 2024; Lind and Lotterhos 2025b) or in some cases, to improve the accuracy (Gain et al. 2023). These findings highlight the importance of investigating the limitations of genomic offset across different species. Key unresolved questions include the role of genetic structure in shaping the variability of genomic offset patterns, the identification of fitness traits that best capture local adaptation, and the influence of environmental variable selection and sampling scheme on projection accuracy.

Our study focuses on black spruce, a widespread boreal species extending from Alaska to Newfoundland and Labrador (Burns and Honkala 1990). This dominant component of Canadian boreal forests displays adaptation to variable soil and climate conditions, ranging from poorly drained bogs to the northern treeline in northwestern Canada, where it can be found in cold, dry climates and on permafrost‐underlain soils. Historically, black spruce has reached its current range from at least two to three distinct glacial refugia situated in the south after the retreat of the Laurentide ice sheet (Jaramillo‐Correa et al. 2004; Gérardi et al. 2010 ). Studies using mitochondrial, cytoplasmic, and nuclear markers have identified three main genetic clusters, roughly corresponding to the West, Central and East regions of Canada (Gérardi et al. 2010; Prunier et al. 2012; Girardin et al. 2021). These findings suggest that current distribution resulted from multiple expansion events from southern refugia and indicate that the species range is composed of partially independent genetic pools within which climate adaptation occurs (Prunier et al. 2012).

The impact of climate on the growth of black spruce has been extensively studied, particularly using dendroecological data. The interplay between temperature and precipitation determines water availability, making drought stress one of the most important factors limiting growth and productivity across the species range (Girardin et al. 2024). Compared to other species such as jack pine, black spruce generally exhibits poor recovery following drought stress (Hogg et al. 2013; Price et al. 2013; Marchand et al. 2021, 2025). Although higher temperatures can enhance growth in some northwestern populations, they more often exacerbate the drought conditions and have negative effects on growth (Walker et al. 2015; Sniderhan et al. 2021). In the more humid eastern portion of the range, growth is mostly limited by cold temperatures in the north, whereas in the south, warmer conditions can increase water availability constraints and respiration costs (Girardin et al. 2016).

Taken together, forecasts generally predict that increasing temperatures and droughts will lead to higher vulnerability especially of western and central North American black spruce populations where precipitation is the limiting factor (Lesven et al. 2024). While northeastern populations can potentially experience growth increase in the near future, this effect is expected to be transient (D'Orangeville et al. 2018). So far, only a few studies have explicitly considered genetic variation of black spruce, focusing on modeling productivity and resistance to extreme drought events in the context of assisted migration along multiple climate gradients (Girardin et al. 2021; Robert et al. 2024, 2025). These studies showed that genetic divergence between black spruce genetic clusters influences phenotypic response to climate that can accumulate over time (Girardin et al. 2021; Robert et al. 2024). Models suggest that northernmost populations may experience a transient increase in growth due to reduced cold limitation. However, over time, all populations are projected to exhibit declines in biomass, with the West cluster showing lower growth relative to the Central and East clusters (Robert et al. 2024). It is important to note though that other climate‐mediated factors can exacerbate or counteract these projections. These include outbreaks of insects such as eastern spruce budworm (Bellemin‐Noël et al. 2021), reduced post‐fire recovery (Baltzer et al. 2021), loss of protective snow cover (Marquis et al. 2022), and shifts in tree species composition which can alter interspecific competition dynamics and reshape soil symbiotic communities. In addition, changes in bud phenology, where higher temperatures can lead to earlier bud break in the spring, may increase the risk of late‐spring frost damage (Rossi 2015; Guo et al. 2022; Mura et al. 2022). While this study focuses specifically on direct effects of climate, common garden experiments in black spruce provide an ideal framework to assess how genome‐wide variation can improve predictions of tree response to changing climatic conditions.

Here, we examined > 1400 black spruce trees for genotypes and 17 phenotypic traits originating from 66 populations and grown in four common gardens. We leveraged nearly 30,000 DArTseq single nucleotide variants, in combination with climate and phenotypic information to validate genomic offset projections across the species range. Our main objectives were: (i) to determine fine‐scale genetic structure and the distribution of genetic diversity across the range of black spruce, (ii) to validate genomic offsets using a space‐for‐time substitution based on the phenotypes produced in the common garden experiment, and (iii) to project genomic offset of black spruce under future climatic conditions. Our extensive validation of genomic offset in a species with a pronounced genetic structure provides valuable insights for informing future forestry management strategies in the face of changing climate.

## 2 Materials and Methods

### 2.1 Sample Collection and DNA Extraction

Samples were collected between 2014 and 2019, from 70 provenances of black spruce, to which we refer to as populations throughout the text. A total of 2237 individuals were sampled, spanning most of the species' natural range in Canada and the USA (Figure 1A, Table S1, Dataset S1). These samples were gathered from common gardens located in Chibougamau (CH, Quebec), Acadia (AC, New Brunswick), Mont‐Laurier (ML, Quebec), Peace River (PR, Alberta), and Valcartier (VL, Quebec), all of which were initially established in 1974 or 1975 as part of the Range‐Wide Provenance Study, with seeds sown in 1970 (Morgenstern 1978; Table S2). Only a small fraction of samples (*n* = 37, nine of which remained after filtering) was collected from VL and used exclusively for genotyping. Additionally, we collected samples from 37 red spruce ( *P. rubens* Sarg.) trees, from various populations within the allopatric zone of the species (Dataset S2). Red spruce is known to hybridize with black spruce; we therefore included it in our analysis to account for genetic admixture between the two species.

**FIGURE 1:**
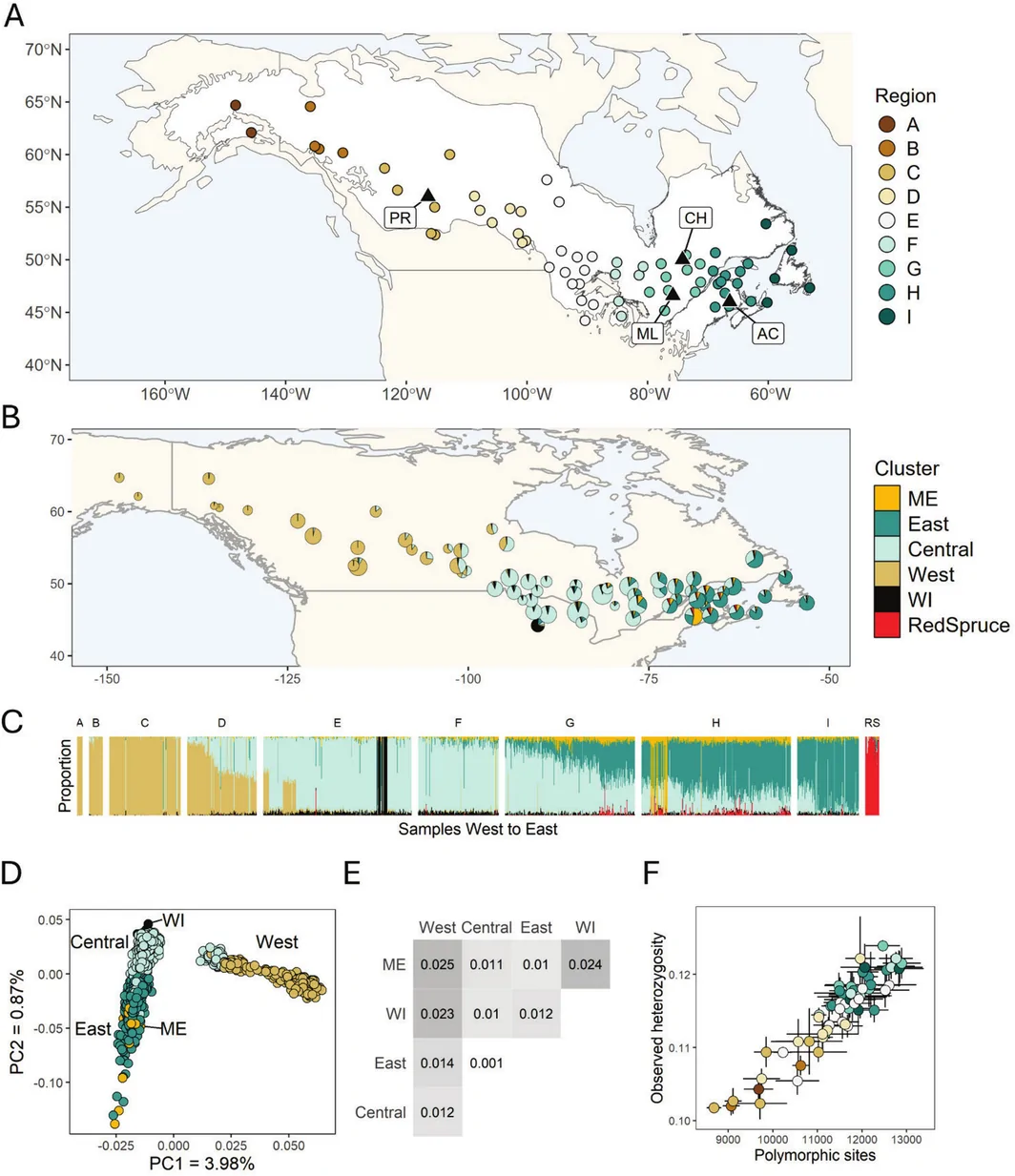
Sampling and population structure of black spruce. (A) Sampling and location of common gardens. Dots indicate population locations, colored by geographic regions delineated at equal intervals along the longitudinal axis. Black triangles indicate the location of common gardens (AC, Acadia; CH, Chibougamau; ML, Mont‐Laurier; PR, Peace River). White area shows the distribution of black spruce. (B) Pie charts show mean genetic ancestry proportions per population, with pie size proportional to population sample size. (C) Admixture analysis showing ancestry proportions of samples at *K* = 6, including red spruce (RS). (D) Principal component analysis computed on 29,972 biallelic SNPs. Colors correspond to genetic clusters depicted in panel B. (E) Pairwise *F*_ST_s matrix among five genetic clusters of black spruce. (F) Observed heterozygosity and number of polymorphic sites across populations. Populations are colored by geographical regions depicted in panel A.

The DNA required for sequencing was extracted from 30–50 mg of either frozen needle tissue (for the CH, ML, and VL sites) or cambial tissue (for AC and PR sites) with a Nucleospin 96 Plant II kit (Macherey‐Nagel, Bethlehem, PA) using the centrifugation processing protocol with a cell lysis step with PL2 buffer for 1 h at 65°C. Cambium (with phelloderm) tissue was obtained from bark samples collected with a 1 cm diameter punch sterilized with 70% alcohol between each sample. Prior to extraction, all tissues were ground to powder with a Mixer Mill MM300 (Retsch GmbH, Haan, Germany) after being plunged into liquid nitrogen for 2 min (this process was repeated twice).

### 2.2 Sequencing and Genotyping

For single nucleotide polymorphism (SNPs) discovery, we employed the DArTseq method, which shares similarities with genotyping‐by‐sequencing (GBS) but includes a complexity reduction step targeting low‐copy sequences within the genome. Our procedure involved digesting genomic DNA with PstI and MseI restriction enzymes, ligating barcoded adapters, amplifying the resulting products via PCR, and sequencing them using a HiSeq 2500 system. This entire process was conducted by Diversity Arrays Technology, headquartered in Bruce, Australia, as described by Kilian et al. (2012). Our SNP analysis focused exclusively on biallelic SNPs for subsequent investigation. The DNA plates containing extracted samples were sent and processed in three consecutive years: 2018, 2019, and 2020, referred to herein as batches 1, 2, and 3 (Dataset S1). Genotypes were generated separately for each of these three batches, and at the conclusion of the project, all sequences produced were merged and clustered together to create the final dataset for analysis.

### 2.3 Data Processing

#### 2.3.1 Genetic Data

SNPs were generated from ~70 nt sequence clusters by setting different sequence identity thresholds producing four independent sets of variants. After checking SNP genotyping rates and ensuring no overlap among sequence tags from the four files, SNP sets resulting from the three lowest clustering thresholds were combined. SNPs were filtered separately for the black spruce and red spruce samples. We filtered out variants with more than 50% of missing genotypes, heterozygosity above 50%, and the minor allele frequency (MAF) below 1%. We ran admixture analysis to determine population structure and at the same time identify potential mislabelled samples. The number of genetic clusters was determined with ADMIXTURE v1.3.0 (Alexander et al. 2009) using all black and red spruce individuals. First, genotypes were saved to plink format using dartR package v2.9.7 (Mijangos et al. 2022). Genetic ancestries were assigned for the *K* number of clusters, where *K* ranged between 2 and 15. Five‐fold cross‐validation error was estimated with each run. The cross‐validation error curve flattened starting with *K* = 4, pointing to three main black spruce genetic clusters (West, Central, East) and one red spruce cluster. Several samples showed assignment to groups from distant clusters, implying potential mislabelling of samples. For the final dataset, we removed most dubious samples, including five samples in the region A and B (Alaska) assigned to the Central or East genetic cluster, and 1 sample from the region I (Newfoundland) assigned to the West cluster. In addition, we removed samples with a call rate below 85%, retaining 1467 black spruce samples from 66 populations (mean of 22 samples per population, range 1–49). A dataset of unique black spruce variants consisted of 29,972 biallelic SNPs.

#### 2.3.2 Climate Data

We used the BioSIM v11 (Fortin et al. 2022) to simulate daily weather at the populations' origins and that of the common gardens for the period 1961–1990. We then calculated 43 climate variables representing temperature, precipitation and the interplay between them, such as climate moisture index (CMI), at the annual or seasonal scale (see Table S3, Dataset S3, for a complete list of variables and the climate data aggregated over the full period for all populations and five common gardens). We obtained simulated daily climate data between 2021 and 2100 for the three emission scenarios (shared socio‐economic pathways): SSP2‐4.5, SSP3‐7.0, and SSP5‐8.5, using an ensemble of 13 climate models from the Ouranos ESPO‐G6‐R2v1.0.0 dataset (Lavoie et al. 2024). Annual and seasonal climate variables corresponding to a subset of those obtained for the past period (a total of 23) were calculated from daily climate data. Mean annual CMI for the future period was obtained by importing daily variables to BioSIM. To ensure that variables obtained with BioSIM and those estimated from PAVICS datasets were comparable, we visually inspected the temporal distributions of annual climate means per population to confirm that means calculated for past and future periods followed a continuous trend and retained 14 of them (CMI, mayMinT, TP, MAT, MMinT, MMaxT, sumTP, WSTP, winMT, sprMT, sumMT, fallMT, MCMT, MWMT). For clarification, we refer to these 14 climate variables, collectively with photoperiod (dPP), as the “small set of climate variables”.

#### 2.3.3 Phenotypic Data

Population means and standard errors of all traits used as a proxy for fitness are given in Dataset S4. Accumulated biomass and wood density (PR: *n* = 397, ML: *n* = 612, CH: *n* = 507, AC: *n* = 481) were obtained from wood cores as described in Robert et al. (2024). Tree total aboveground accumulated biomass (kg) was calculated as the sum of annual biomass between years 1980 and 2015 (ABtot), in addition to eight nonoverlapping five‐year intervals between 1980 and 2020 (AB5). Wood density was reported as the average wood density for the ring from the year 2015. Height in cm and diameter at breast height (DBH) in cm were measured in 2019 (PR: *n* = 400), 2015 (ML: *n* = 667), 2016 (CH: *n* = 451), and 2017 (AC: *n* = 492). Survival was reported as the % of surviving trees per population in 2011 (PR: *n* = 26), 2007 (ML: *n* = 42), 2006 (CH: *n* = 38), and 2003 (AC: *n* = 40). Four drought‐related indices to extreme drought events (sensu Lloret et al. 2011) were calculated from the wood cores for three drought years at AC, two at CH, five at ML, and four at PR, and averaged for each site (Robert 2026). These indices included resistance (Rt), growth during the year of the drought over the average growth of the 2 years prior to the drought (higher ratio means higher resistance); recovery (Rc), the average growth of the 2 years after the drought over the growth during the year of the drought (higher ratio means higher recovery); resilience (Rs), the ratio of growth after the drought event over the growth before the event (higher ratio means higher resilience); and relative resilience (Rr), resilience minus resistance (higher ratio means higher relative resilience).

To obtain population‐level trait estimates while accounting for the experimental design of the common gardens, we fitted the mixed model to each trait except for survival:

$$\text{individual tree trait}\sim \text{block}+\left(1|\text{population}\right)+\left(1|\text{block}:\text{population}\right).$$

We removed the estimated block effect and the BLUP (best linear unbiased prediction) associated with the population × block interaction from each individual observation. This procedure removes variation due to differences among blocks and to population‐specific within‐block deviations, while retaining variation among populations. The corrected values were then averaged for each population within each common garden. ABtot and AB5 were first log transformed. Blocks with less than 10 individuals were excluded.

### 2.4 Population Structure Analyses

Population structure was determined using an ADMIXTURE program, as described above, and principal component analysis was used to visualize ordination of genetic clusters. Genotypes of the black spruce individuals were saved to gds format using dartR package v2.9.9.5 (Mijangos et al. 2022). PCA was run using package SNPRelate v1.45.0 (Zheng et al. 2012).

To investigate spatial structure, we retained 63 black spruce populations with at least five individuals. Weir and Cockerham estimate of *F*_ST_ between pairs of populations was calculated with assigner v0.7.0 (Gosselin et al. 2019) on a random subset of 1000 SNPs. In this analysis, genotypes were transformed to a tidy format using radiator v1.4.0 (Gosselin 2020). Geographic distances (km) between populations were derived from spatial coordinates converted to a spatial sf object using a WGS 84 coordinate reference system from sf v1.1‐0 (Pebesma 2018). To test for isolation by distance, a Mantel test was performed by correlating matrices of genetic distances (*F*_ST_/(1 − *F*_ST_)) and geographic distances with package vegan v2.7‐3 (Oksanen et al. 2025), with 9999 permutations, and the Spearman's rank correlation.

In parallel, spatial autocorrelation and the relationship between genetic and geographic structure were assessed using Moran's eigenvector maps (MEMs). First, a spatial weighting matrix was created for populations with parameters “type = 6, *k* = 8” using adegenet v2.1.11 (Jombart 2008) and spdep v1.4‐2 (Bivand and Wong 2018). MEMs were then computed with package adespatial v0.3‐29 (Guénard and Legendre 2022).

For spatial PCA (sPCA), we estimated allele frequencies within populations with dartR package v2.9.9.5 (Mijangos et al. 2022) and removed SNPs with missing values in any of the populations. sPCA was run with package adegenet v2.1.11 (Jombart 2008), using a spatial weighting matrix estimated above. Finally, Moran's coefficient was calculated with package adespatial v0.3‐29 (Guénard and Legendre 2022) to assess whether genetic variation captured by the principal components is spatially autocorrelated.

Pairwise *F*_ST_s between clusters were estimated in the same way as for populations. Individuals were assigned to one of the five clusters identified with admixture analysis at *K* = 6, excluding red spruce (West, Central, East, WI, and ME). Each individual was assigned to a cluster based on the dominant ancestry assignment. Analysis of molecular variance (AMOVA) was conducted at three hierarchical levels: cluster, population, and individual. Only populations with at least 5 individuals were retained. AMOVA was estimated with poppr v2.9.8 (Kamvar et al. 2014). Heterozygosity was calculated individually for each population, using the original dataset only filtered for variants not sequenced in more than 50% of samples, while retaining rare alleles. We focused on 60 populations represented by at least seven individuals and randomly selected seven genotypes five times. For each subset, we removed any missing and monomorphic sites and calculated observed heterozygosity as the number of heterozygous SNPs for each individual divided by all sites and averaged across samples in each population. Expected heterozygosity was calculated for each population as the sum of squared frequency of allele 1 and 2 in each site, averaged across sites and subtracted from 1.

### 2.5 Spatial Associations Between Genotypes and Climate

To visualize clustering of samples by climate, we ran PCA on 43 scaled and centered climate variables using the prcomp function from the base R package stats. To estimate the extent to which the distribution of genetic diversity is correlated with climate, we conducted a partial redundancy analysis (pRDA) by fitting three main climate principal components (PCs), three main Moran eigenvectors (MEMs), and two main PCs from genetic diversity ordination to a matrix of population allele frequencies. pRDA models were fitted to determine the impact of genetic, spatial, and climate explanatory variables, one class (spatial, genetic, climate) at a time while controlling for the remaining variables. Models' adjusted *R* ^2^ were estimated and the significance of correlation of each class of variables with genetic variation was tested with a permutation test. In addition, pRDA was conducted at the genetic cluster level, after prior recalculation of two main PCs from genetic diversity ordination and three main MEMs. All functions are found in the vegan package v2.7‐3 (Oksanen et al. 2025).

To determine which climate variables are best associated with genetic variation, we ran Gradient Forest (Ellis et al. 2012; Fitzpatrick and Keller 2015) using 43 climate variables and 3 MEMs depicting spatial patterns of distribution. First, we used the allele frequency matrix generated in previous steps for 63 populations, removed any nonvariant markers, and selected a random subset of 1000 markers. We fitted the model using the gradientForest package v0.1‐37 (Ellis et al. 2012), with 500 bootstrapped trees and a correlation threshold of 0.5. Variables were ranked based on the mean raw accuracy importance.

### 2.6 Associations Between Phenotypes and Climate

To investigate local adaptation to climate, a Spearman's rank correlation was calculated between phenotypic traits and climate transfer distance while accounting for spatial autocorrelation using modified *t*‐test from the R package SpatialPack v0.4‐1 (Vallejos et al. 2020). *p*‐values were corrected for multiple testing with the Benjamini‐Hochberg procedure at a false discovery rate of 0.05. Climate transfer distance was calculated as the Euclidean distance of each population to its origin in climate space defined by four major PC axes of a Principal Component Analysis based on 43 climate variables. To calculate correlations, populations with fewer than four trait values were removed.

### 2.7 Testing Genomic Offset With Common Gardens

We used common garden experiments to test the accuracy of genomic offset projections. In each common garden, models were trained using population‐specific climate variables and allele frequencies, and genomic offsets were then projected to climatic conditions of the common gardens (Figure 3). As a measure of model performance (or genomic offset projection accuracy), we calculated Spearman's rank correlation between genomic offsets that serve as proxies of population maladaptation to common garden conditions and population‐level phenotypic trait means that serve as proxies for fitness. More negative correlation coefficients were interpreted as indicating better model performance.

#### 2.7.1 Defining Training and Test Sets

To compare population genomic offsets with phenotypic traits, distinct sets of samples were used for model training (training dataset) and for phenotypic evaluation (test dataset), thereby avoiding bias associated with testing model accuracy on the same genotypes used for training (Figure 3). Sample assignment to each dataset followed a structured procedure. Samples with only phenotypic data available, due to exclusion of their genotypes during quality filtering, were assigned to the test dataset, while all remaining samples were initially allocated to the training dataset. We then ensured that each population was represented by at least three individuals in the test dataset for reliable estimation of mean phenotypic traits and at least five individuals in the training dataset for model fitting. When necessary, samples were transferred from the training dataset to the test dataset for a given population, provided that at least five individuals remained in the training dataset. In addition, two populations containing individuals assigned to the ME or WI genetic clusters were excluded from model validation to minimize potential confounding effects associated with these genetically distinct groups. In AC, many genotypes were missing due to low‐quality data, resulting in test datasets that contained more populations and individuals than the corresponding training datasets.

The selection of training and test datasets, as well as subsequent analyses, was performed separately for each trait, as the number of samples and populations varied among them. The numbers of populations and individuals per common garden and trait included in the training and test datasets are provided in the Tables S4 and S5.

To investigate the behavior of the genomic offset projection models, we explored seven aspects related to the input data and model training and their effects on model performance (Figure 3).

##### 2.7.1.1 Common Gardens and Phenotypic Traits (Factor 1)

All models were trained separately for each common garden using its corresponding dataset (Figure 3A). As proxies for fitness‐related phenotypic traits in the test datasets, we used population means of 16 traits, excluding survival, adjusted as described above and averaged over test dataset samples only. The traits included: height, total accumulated tree biomass (ABtot), five‐year accumulated biomass (AB5) measured over eight intervals, diameter at breast height (DBH), wood density, resistance (Rt), recovery (Rc), resilience (Rs), and relative resilience (Rr).

##### 2.7.1.2 Marker Types (Factor 2)

We tested the impact of eight different marker types on genomic offset projections, including genome‐wide and climate‐associated SNP markers (single nucleotide polymorphisms). Genome‐wide markers included all available markers (~29 K), a random subset of 100 (0.1 K) or 1000 (1 K) markers, as well as random subsets of 100 or 1000 markers for which no minor allele frequency filter was applied (0.1 K‐lf, 1 K‐lf). Climate‐associated markers included outliers detected with LFMM, RDA, and RDA controlling for population structure (RDA‐struct).

###### 2.7.1.2.1 Genome‐Wide Markers

Genotype datasets were first prepared from the samples present in the training datasets by calculating allele frequencies per population separately for each common garden, while removing any invariant markers, markers with missing data, or low‐frequency markers (minor allele frequency < 0.05). Low‐frequency marker datasets (0.1 K‐lf and 1 K‐lf) were derived from the raw data and subjected to the same filtering pipeline as the standard marker sets, with the sole exception of the minor allele frequency threshold.

###### 2.7.1.2.2 Climate‐Associated Markers

We used a dataset with all available markers (~29 K) combined across common gardens to detect markers showing associations with distinct climate axes (precisely with the three main PC axes on 43 climate variables). Three tests were performed: LFMM, RDA, and RDA with correction for population structure. LFMM v2 was run with LEA v3.24 (Gain and François 2021), using *K* = 3 latent factors (corresponding to three black spruce genetic clusters). *p*‐values were calculated to test the significance of associations between each marker and individual effects of three climate PCs. *p*‐values were then adjusted using Bonferroni correction for multiple testing. RDA was conducted with vegan v2.7‐3 (Oksanen et al. 2025), first with the three climate PCs as only explanatory variables, and second by additionally conditioning for population structure using the two main axes from genetic variation ordination. Marker *p*‐values were obtained by calculating Mahalanobis distance of each variant from the centroid of RDA marker loadings and comparing it with a chi‐squared distribution, following the approach described by Capblancq et al. (2018), and subsequently adjusting with Bonferroni multiple test correction. To estimate the amount of genetic variation represented by the selected list of outliers, observed heterozygosity was calculated. Heterozygosity per population was estimated on a combined set of all available genotypes from the four gardens excluding variants with minor allele frequency < 0.05.

##### 2.7.1.3 Genomic Offset Models (Factor 3)

###### 2.7.1.3.1 Gradient Forest

Gradient Forest was run using the R package gradientForest v0‐1.37 (Ellis et al. 2012), with 500 trees and a correlation threshold of 0.5. Genomic offset was defined as the Euclidean distance between vectors of allelic turnover functions projected for each population under its native climate and under the climate conditions of the common garden. Climate variables were summarized using the first three main PC axes derived from 43 climate variables.

###### 2.7.1.3.2 RDA (Redundancy Analysis)

In parallel, RDA was performed to train the model with the vegan package v2.7‐3, using the same three climate PC axes described above. Genomic space under native and common garden climatic conditions was predicted using the predict() function and the argument type = “lc”. Genomic offset was calculated as the Euclidean distance between vectors of the three RDA axes for each population under native and garden climate, weighted by the relative importance of each climate axis.

##### 2.7.1.4 Training Sensitivity and Validation Stability (Factor 4)

First, we compared model performance between the training and test datasets. Then, we explored the impact of downsampling of the training or test datasets on model performance in three ways. We systematically removed: (i) individual populations from the training dataset, leaving between 6 and 20 populations (with a step of 2) randomly selected in five draws, (ii) individual trees from populations from the training dataset, leaving between a maximum of 2 to 10 trees per population (with a step of 2) randomly selected in 10 draws, and (iii) individual trees from populations from the test dataset, leaving between a maximum of 2 to 10 trees per population (with a step of 2) randomly selected in 10 draws. All models were trained using 1000 random markers.

##### 2.7.1.5 Stratified Validation (Factor 5)

We validated model performance by calculating offset‐trait correlations for all tested populations and separetely for populations belonging to each of the three main genetic clusters (West, Central or East). Each population was assigned to one of the three clusters based on ancestry coefficients derived from admixture analysis. Specifically, individuals were first assigned to a genetic cluster, and populations were subsequently assigned using a majority rule approach.

##### 2.7.1.6 Model Transferability (Factor 6)

We investigated whether: (i) regional models, trained independently within each genetic cluster, improve predictions by capturing local adaptive gradients, and (ii) models trained in one genetic cluster could predict fitness in another. Because each genetic cluster occupies a distinct climate niche, this approach effectively assesses model transferability to novel environments. Genetic clusters were defined as described above. Models were trained as described above, using training datasets of 1000 random markers and three main climate PC axes as predictors.

##### 2.7.1.7 Climate Predictor Sensitivity (Factor 7)

To test the impact of the choice of climate variables on model performance, we trained models using two sets of climate variables: (i) the three main principal component (PC) axes derived from 43 climate variables, and (ii) three uncorrelated climate variables (dPP, CMI, and fallMT) selected based on their Gradient Forest importance rankings. These three variables were selected out of a candidate pool of 15 variables from the small set of climate variables to ensure consistency with the variables available for future climate projections.

#### 2.7.2 Model Validation Calculation and Consistency

To validate genomic offsets, Spearman's rank correlation was calculated if at least five populations had estimates of both genomic offset and mean trait value. We also ensured that at least five trees per population were present in the training dataset and three trees per population were present in the test dataset. 95% confidence intervals were computed with 1000 bootstraps.

Spearman rho coefficients between fitness and climate transfer distance were very similar whether or not spatial autocorrelation was accounted for. For consistency, we therefore report all validation results without correction for spatial autocorrelation. To investigate congruence among genomic offset projections from different models, we calculated Pearson's correlation coefficients.

### 2.8 Genomic Vulnerability of Black Spruce to Future Climate

Future simulated annual means of a small set of climate variables (CMI, mayMinT, TP, MAT, MMinT, MMaxT, sumTP, WSTP, winMT, sprMT, sumMT, fallMT, MCMT, MWMT, dPP) were averaged across four 20‐year periods: 2021–2040, 2041–2060, 2061–2080, and 2081–2100. To illustrate the direction and magnitude of projected climate change across emission scenarios, regions, and time periods, we classified past and multiple future population climates into a limited number of climate types. Climate variables were scaled and clustered using *K*‐means (KMeans() scikit‐learn v1.7.0), testing 1 to 25 clusters. For each value of *K*, initial cluster centres were selected using a single run of the greedy k‐means++ algorithm (n_init = “auto”), which spreads initial centres across the data. The optimal number of clusters was selected using the elbow method (yellowbrick v1.5), which identifies the point beyond which adding clusters no longer substantially decreases the distortion score (the sum of squared distances of data points to their cluster centre). The method partitioned all data points into 10 clusters, which were visualized on the two main principal components. The resulting climate types were used solely as a visualization tool to summarize similarities among historical and projected climates.

Gradient Forest was run as described above on a subset of 1000 randomly selected SNPs and allele frequencies calculated for each population, including those from genetic clusters ME and WI, to span the full range of black spruce genetic diversity. SNPs with anymissing frequency estimates or no variation were excluded. Genomic offsets were calculated for the four 20‐year periods and SSP2‐4.5 emission scenario. Two models were trained: (i) a model trained on all populations and the three main PCs derived from a small set of climate variables to project genomic offsets in the Central and East clusters, and (ii) a model trained on populations from the West cluster and a subset of three selected climate variables (dPP, CMI, and fallMT) to project genomic offsets in the West cluster. Although we used two different climate datasets (BioSIM and Ouranos) to maintain consistent climate variables across past and future periods, future projections would ideally be based on a single climate dataset. Otherwise, systematic differences between datasets may be confounded with projected climate change, potentially biasing genomic offset estimates, particularly in regions or periods where projected climate changes are relatively small.

Primary filtering analyses were performed using R v4.4.2 (R Core Team 2024), while all other analyses were conducted using R v4.6.2 (R Core Team 2026) and Python v3.12.

## 3 Results

### 3.1 Black Spruce Population Structure

We analyzed 1467 black spruce individuals from 66 populations (Figure 1A, Table S1, Dataset S1). We identified 3 main genetic clusters of black spruce: West, Central and East, with most samples showing mixed ancestry between West and Central or Central and East clusters (Figure 1B,C). PCA showed similar results, with the 1st PC separating samples from cluster West, and the 2nd PC spreading samples from clusters East and Central, but with no clear separation (Figure 1D). At higher values of *K*, the admixture analysis distinguished two additional subclusters: WI (samples from a population in Wisconsin) within the Central cluster, and ME (samples from a population in Maine) within the East cluster (Figure 1B,C). *F*_ST_ was 0.012–0.014 between the cluster West and the clusters East or Central, about 10 times higher than between clusters Central and East (*F*_ST_ = 0.001, Figure 1E). Clusters WI and ME were most differentiated from cluster West (*F*_ST_ = 0.023 and 0.025, respectively) and from each other (*F*_ST_ = 0.024). AMOVA showed low but significant variance between clusters (% variance between clusters = 4.6%, *p* < 0.01, Table S6).

Black spruce populations showed a significant pattern of isolation by distance (Mantel's *R* = 0.78, *p* < 1 × 10^−4^, Figure S1). The inferred genetic clusters were geographically separated (Figure 1B), and the two main axes of spatial autocorrelation (Moran eigenvector maps, MEMs) overlapped with the distribution of these clusters (Figure S2), indicating that geographic distance is an important contributor to genetic differentiation among black spruce populations. The two main axes of spatial PCA, confirmed the separation of West cluster and a split between the Central and East clusters (Figure S3), lending support to the presence of three genetic clusters. The gradual transition in ancestry coefficients between neighboring genetic clusters is therefore consistent with the strong spatial genetic structure observed across the species range.

Observed heterozygosity ranged between 0.10 and 0.12 per population (Figure 1F). Most populations from the West cluster showed lower heterozygosity and fewer polymorphic sites. When heterozygosity was measured excluding monomorphic sites within each population, West cluster populations showed higher observed heterozygosity despite a lower number of polymorphic sites (Figure S4), showing that in the West cluster heterozygosity is driven by many monomorphic sites rather than the higher fraction of homozygous sites.

### 3.2 Local Adaptation of Black Spruce to Climate

The three genetic clusters of black spruce occupied distinct climate niches. Population locations were characterized in terms of climate by compiling 43 climate variables averaged across 30 years (from 1961 to 1990, Table S3, Dataset S3). The first two principal components (PCs) summarizing these variables distinguished populations from the West, Central, and East, with eastern locations being the warmest and most humid and western ones being the driest and coldest (Figure 2A). Partial RDA analysis conditioned on genetic structure, spatial structure, and climate variables showed that each class of variables explained only a small proportion of genetic variation on its own (1.8%–3.6% of explained variance, *p* = 0.001, Table S7). Climate alone explained 1.1% of genetic variation (adj. *R* ^2^ = 0.011), corresponding to 2.6% of the explained variance, while most of the explained variation was shared with spatial and genetic structure. This pattern indicates that climate, geography, and demographic history are strongly confounded across the species range, making it difficult to disentangle their individual contributions to genomic variation. To identify which environmental variables were best associated with the distribution of genetic diversity, we ran Gradient Forest on population frequencies estimated for 1000 randomly selected variants. Photoperiod, climate moisture index, precipitation, and fall frost probability were among the most important predictors, besides the main axes of spatial autocorrelation (Figure 2A,B).

**FIGURE 2:**
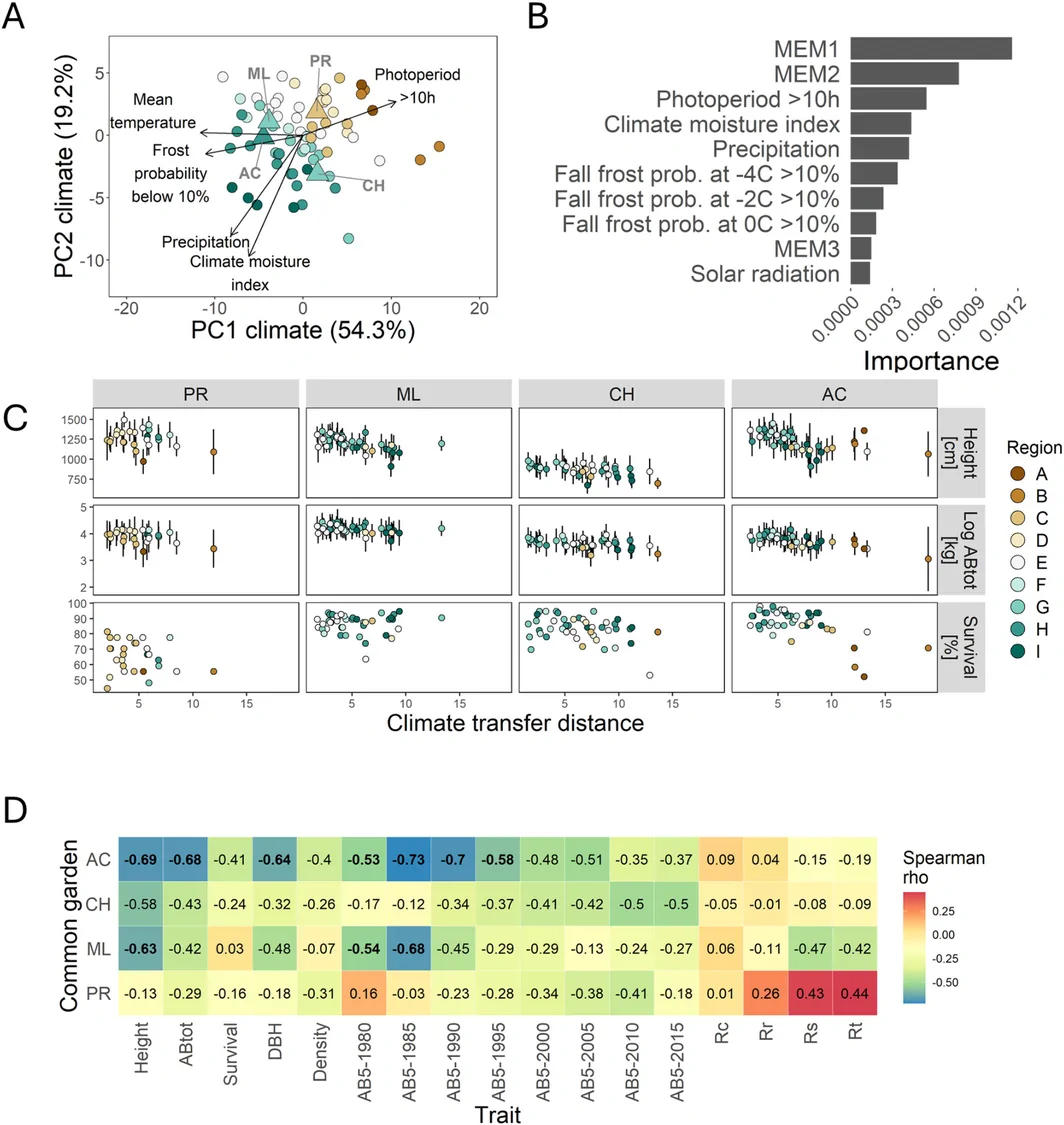
Local adaptation of black spruce to climate. (A) Ordination of populations based on 43 climate variables. Vectors representing five climate variables identified using Gradient Forest are shown as arrows, with lengths scaled by a factor of 12. Locations of four common gardens (PR, ML, CH, AC) are shown by triangles. (B) Importance ranking of the top climate and spatial variables associated with genetic variation across species distribution. (C) Population means with standard deviation of height, accumulated biomass and survival rate plotted against climate transfer distance, defined as the Euclidean distance between the climate of origin of each population and that of the common garden site. (D) Rank correlation coefficients between population means of traits and climate transfer distance. Bold values indicate coefficients that are statistically significant (*p* < 0.05) after Bonferroni correction for multiple testing. ABtot, total accumulated biomass; Rc, recovery; Rr, relative resilience; Rs, resilience; Rt, resistance; AB5, five‐year accumulated biomass. The year following AB5‐ labels indicates the beginning of the five‐year period over which accumulated biomass was estimated. Colors in panels A and C correspond to geographic regions shown in Figure 1A.

Measurements of multiple phenotypic traits in common gardens revealed that black spruce populations originating from climates distant from the garden's conditions tended to show reduced fitness compared to those from similar climates, implying local adaptation to climate. Height, total accumulated biomass (ABtot), and diameter at breast height (DBH) showed the strongest negative correlations with climate distance, consistent with local adaptation, although these correlations were consistently weaker in PR (rho > −0.39, Figure 2C,D). Survival rate and wood density were generally weakly correlated. Five‐year accumulated biomass (AB5) traits showed variable patterns depending on tree age and common garden: with stronger negative correlations observed at later growth stages in CH and PR, and at younger stages in ML and AC (Figure 2D). Also, metrics of drought resilience varied, with weakly negative correlations observed only for resilience (Rs) and resistance (Rt) in AC and ML (Figure 2D).

### 3.3 Testing Genomic Offset With Common Gardens

#### 3.3.1 Common Gardens and Phenotypic Traits (Factor 1)

Using the genomic offset validation framework described in Figure 3, model performance varied across common gardens and traits (Factor 1; Figure 4A, Figure S5). In ML, CH and AC, traits that were more correlated with climate transfer distance also tended to show stronger correlations with genomic offset, although genomic offsets rarely outperformed climate‐based correlations. The highest model performance was observed in AC, despite the training dataset including the fewest populations (*n* = 10), and most test populations being absent from the training set. In contrast, in PR, genomic offsets were either uncorrelated or positively correlated with fitness traits.

**FIGURE 3:**
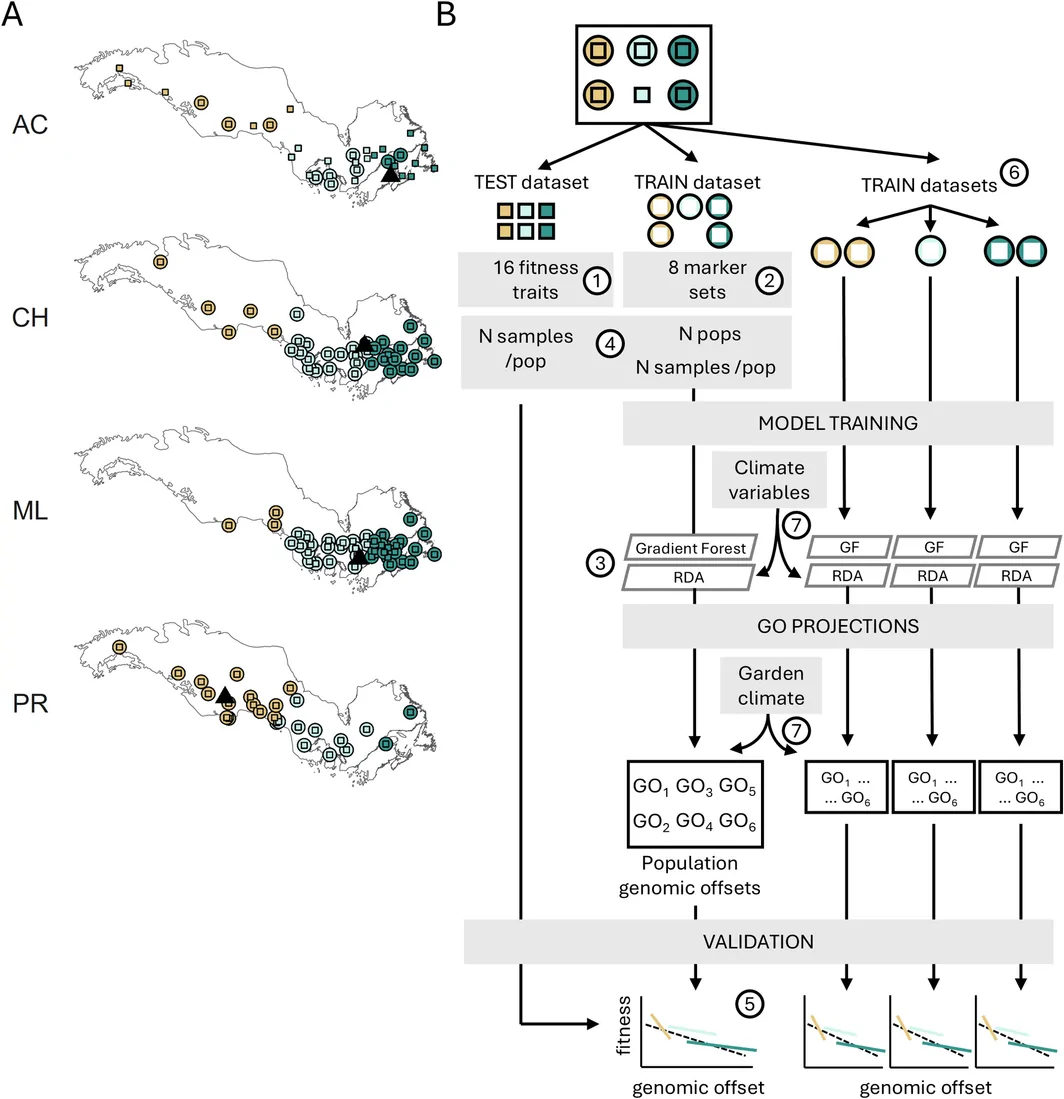
Validation framework for genomic offset projections. (A) Sampling scheme showing populations used for model training (circles) and testing (squares) within each common garden. Populations were included in both datasets, when possible, except when genotype data were unavailable (e.g., AC). Black triangles indicate common garden locations. (B) Workflow used to validate genomic offset projections. For each common garden, samples were partitioned into training and test datasets. Models (Gradient Forest (GF) and RDA) were trained using population allele frequencies and climate variables, and genomic offsets (GO) were projected under common garden climate conditions. Model performance was evaluated by correlating genomic offset with population means fitness traits (Spearman rho), where more negative values indicate better predictive performance. Numbers indicate the seven factors evaluated: (1) fitness traits, (2) eight marker types, (3) two modeling approaches, (4) subsampling sensitivity, (5) stratified validation (population vs. cluster scale), (6) transferability (training at species vs. cluster level), and (7) climate variable selection.

**FIGURE 4:**
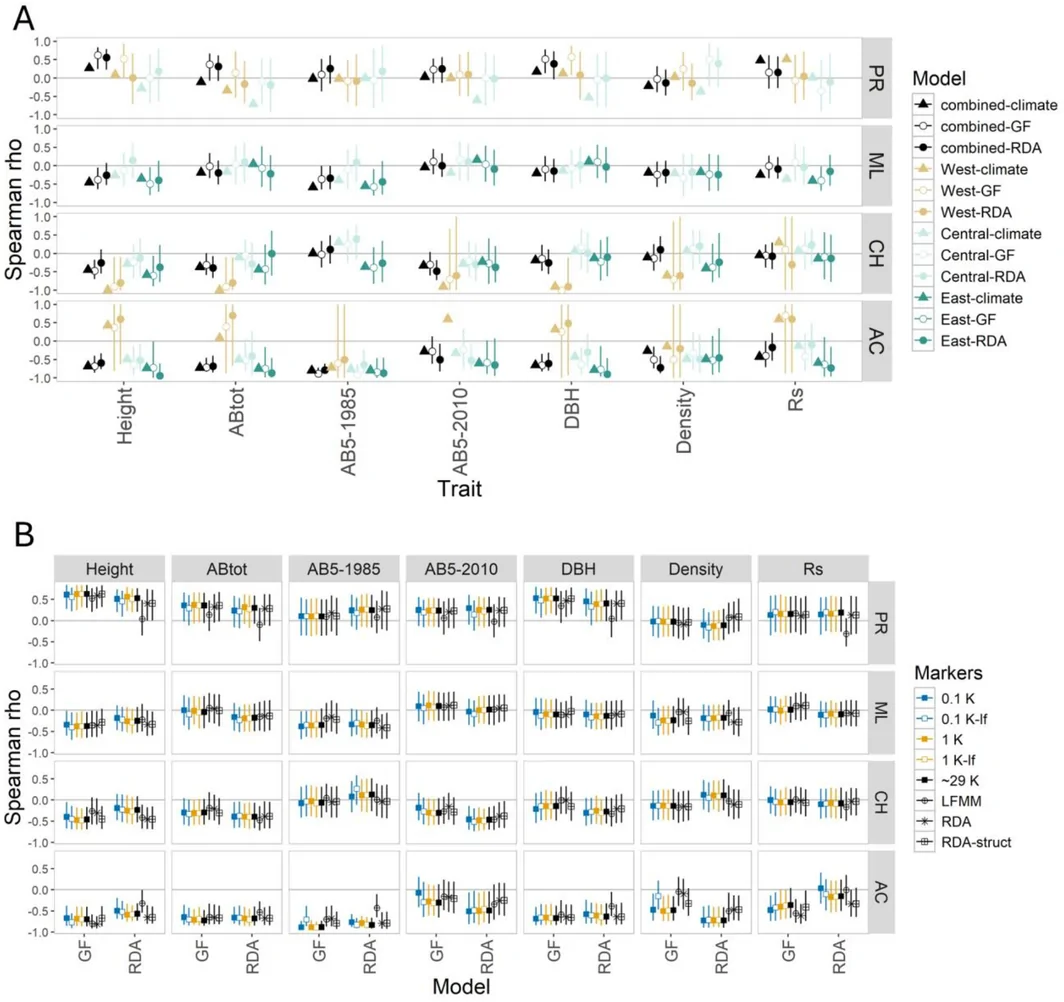
Accuracy of genomic offsets projections across models and marker sets. (A) Comparison of rank correlations between fitness traits and climate transfer distance or genomic offsets from two models (Gradient Forest—GF and redundancy analysis—RDA), when evaluated for all populations (combined) or individual genetic clusters. (B) Comparison of rank correlations between models trained on eight different marker sets, including all populations (combined). The marker types included subsets of 100 (0.1 K), 1000 (1 K) or all available markers (~29 K), subsets including low‐frequency variants (0.1 K‐lf, 1 K‐lf), and markers found to be in association with climate (LFMM, RDA, RDA‐struct).

Across AC, CH, and ML, height and ABtot showed the strongest negative correlations with both climate distance and genomic offsets. Early interval AB5 was more negatively correlated with genomic offsets in AC and ML, whereas later AB5 showed stronger correlation in CH. Overall, genomic offsets were weakly associated with drought resistance traits, although negative correlations were observed for Rs and Rt in AC.

#### 3.3.2 Marker Types (Factor 2)

No consistent differences in model performance were observed when using random markers, all available markers, or subsets of markers associated with three climate PCs, either corrected or uncorrected for genetic structure (Factor 2; Figure 4B, Figure S6). LFMM outliers represented an exception to the general pattern: they improved model performance in PR across most traits, but reduced performance in AC for RDA models and in CH for Gradient Forest models.

Genomic offsets derived from genome‐wide marker sets were highly correlated with each other (Figures S7–S10), but less so with two climate‐associated markers (LFMM and RDA) in both Gradient Forest and RDA models. LFMM outliers also showed the lowest heterozygosity among marker types (Figure S11), suggesting an enrichment in markers with locally fixed alleles.

#### 3.3.3 Genomic Offset Models (Factor 3)

The two modeling approaches, Gradient Forest and RDA, produced broadly similar results, although mean performance varied slightly depending on the garden and trait (Factor 3; Figure 4A, Figure S5). Gradient Forest performed better on average for traits such as height and Rt in AC, whereas RDA showed stronger correlations with wood density in AC or AB5‐1990 in ML.

#### 3.3.4 Training Sensitivity and Validation Stability (Factor 4)

Sensitivity to the training dataset was moderate (Factor 4; Figures S12–S14). Model performance was usually slightly higher when models were trained on the test datasets compared to the training datasets, suggesting reduced predictive accuracy for unseen genotypes. However, these differences were very small. Among the most significant results in AC, model performance was higher when models were trained on the training dataset than on the test dataset for height and AB5‐1985 (Figure S12), most likely because of the smaller number of populations in the test dataset.

The effect of increasing the number of populations in the training dataset varied depending on the garden, model type, and trait (Figure S13). For example, for height, adding populations improved performance in Gradient Forest models (AC, ML, CH) but reduced performance in RDA models (AC, CH). In PR, using fewer populations in the training dataset brought offset‐trait correlations closer to zero.

The effect of increasing the number of individuals per training population also varied, depending on the garden, model type, and trait (Figure S14). For height and ABtot, increasing the sample size had little or no effect or reduced model performance, in particular for RDA.

The influence of population size in test datasets was more evident (Figure S15). Model performance in AC decreased with smaller test population sizes. In ML and CH, performance was more stable and declined primarily for the smallest populations. In PR, where offset‐trait correlations were positive, correlations generally tended towards zero as test population size decreased.

#### 3.3.5 Stratified Validation (Factor 5)

When evaluated at the level of genetic clusters, model performance was not consistent across clusters (Factor 5; Figure 4A, Figure S5), a pattern also observed for climate‐trait relationships. Offset‐trait correlations for the East cluster were negative across all common gardens. In contrast, the Central cluster showed negative correlations only in AC, and the West cluster only in CH. Although discrepancies between climate distance and genomic offset persisted in PR, they were less pronounced for RDA models.

#### 3.3.6 Model Transferability (Factor 6)

For models trained on populations belonging to individual genetic clusters, model performance was similar to that of models trained on the combined datasets (Factor 6; Figure 5, Figures S16 and S17). However, genomic offsets derived from models trained on different clusters were not always strongly correlated. In PR, offsets obtained from models trained on the Central cluster were often poorly correlated with those trained on the West cluster or on the combined dataset (Figures S18 and S19). In CH, strong agreement was observed only between RDA models trained on combined and East, or Central and West datasets. In addition, models performing well in one cluster, did not have equal performance in other clusters or across the species range (Figure 5, Figures S16 and S17). Climate explained a significant part of genetic variation at the cluster level, even though most of it was confounded with genetic and spatial structure (Table S8), indicating that the lack of improvement from cluster‐specific models was not due to an absence of genotype–climate association within clusters.

**FIGURE 5:**
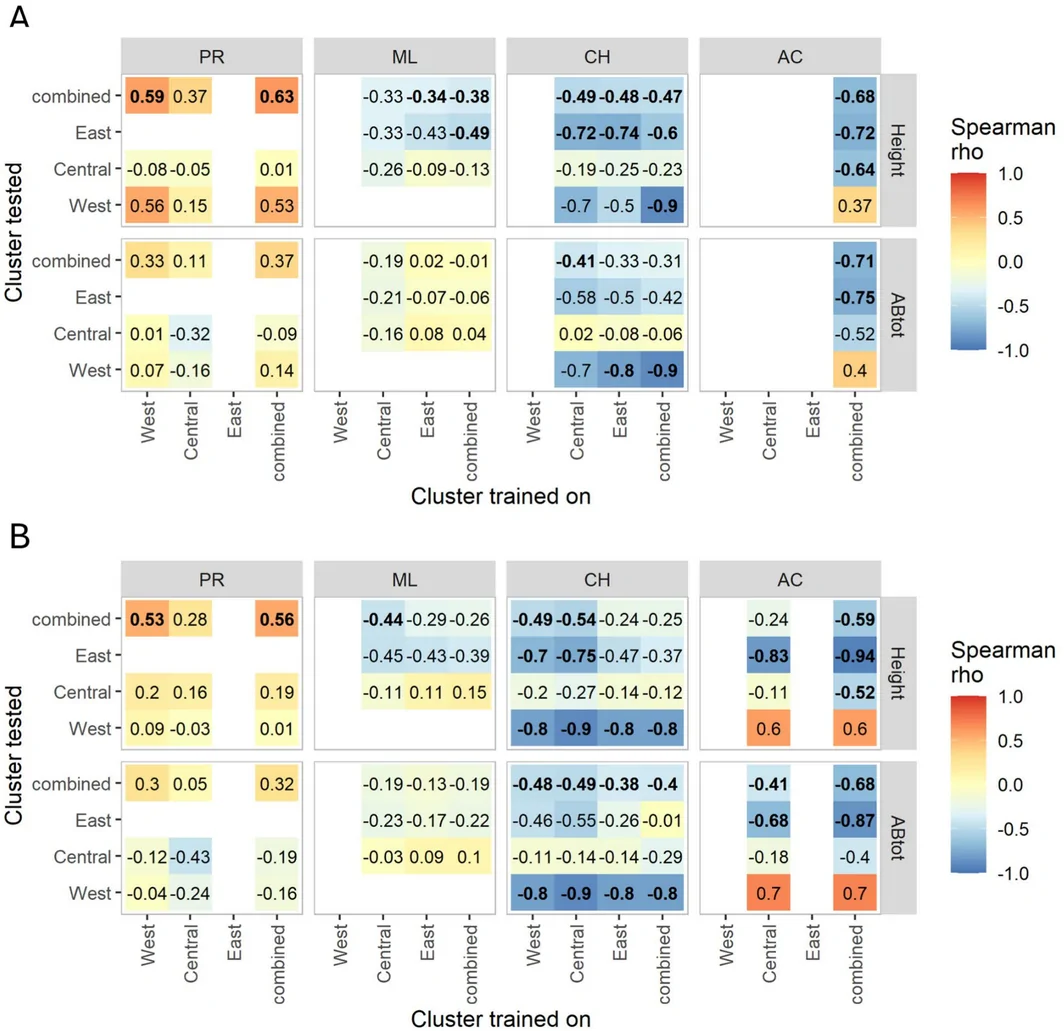
Comparison of models trained and evaluated on combined datasets or individual genetic clusters using three major climate PCs. (A) Genomic offset‐trait rank correlations for Gradient Forest models. (B) Genomic offset‐trait rank correlations for RDA models. All models were trained using 1000 random markers.

#### 3.3.7 Climate Variable Selection (Factor 7)

Model sensitivity to climate predictors varied across gardens (Factor 7; Figure 6, Figures S20–S23). In ML, CH, and AC, model performances generally decreased when using three climate variables instead of three climate PCs, regardless of whether the models were trained on combined datasets or individual clusters only, except for those trained on the West cluster in CH. In contrast, in PR, offset‐trait correlations obtained with Gradient Forest in West clusters improved and were nearly always negative.

**FIGURE 6:**
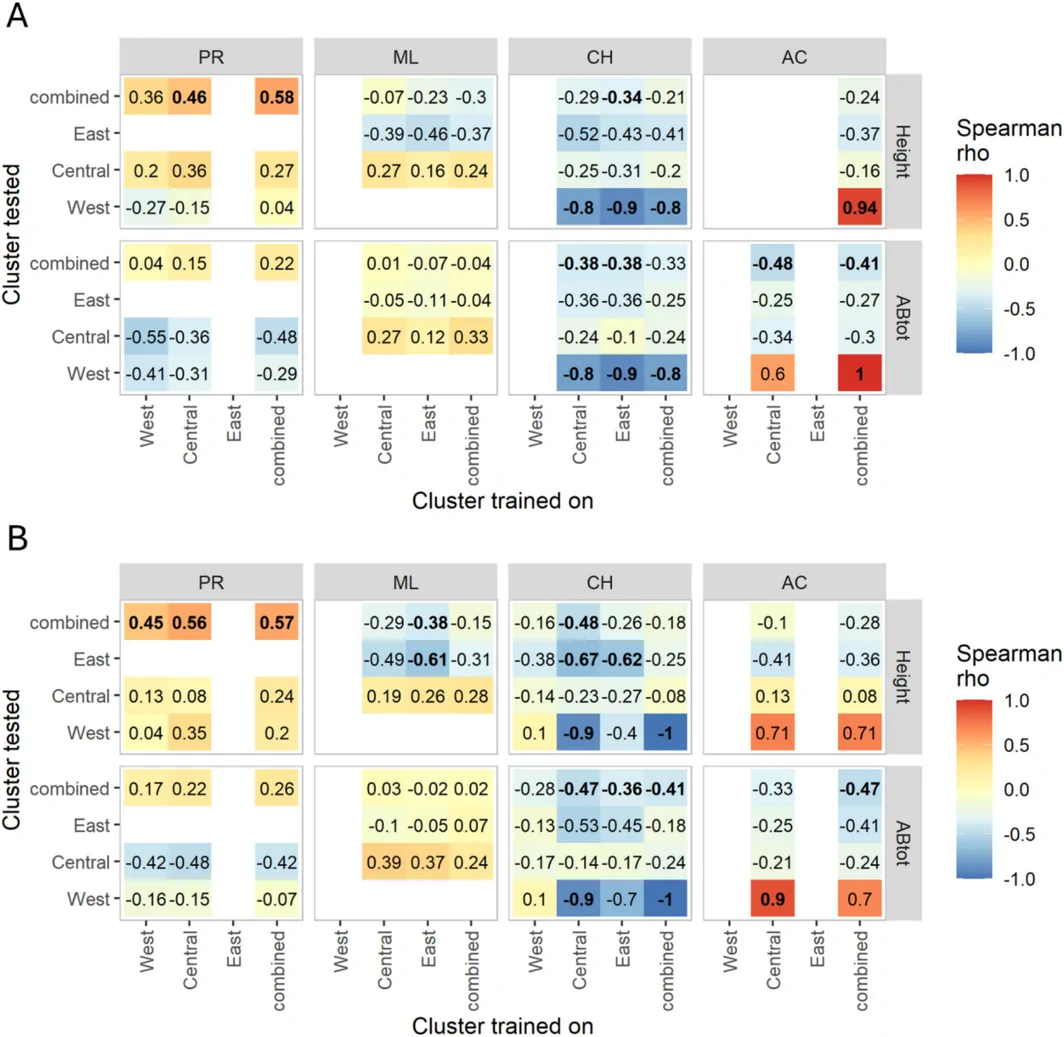
Comparison of models trained and evaluated on combined datasets or individual genetic clusters using three climate variables (dPP, CMI and fallMT). (A) Genomic offset‐trait rank correlations for Gradient Forest models. (B) Genomic offset‐trait rank correlations for RDA models. All models were trained using 1000 random markers.

Genomic offsets of models trained on three PCs vs. three climate variables were weakly correlated, with a mean of 0.55 for Gradient Forest models (range from −0.07 to 0.97) and 0.72 for RDA (range from −0.65 to 0.97; Figures S24 and S25).

### 3.4 Vulnerability of Black Spruce Populations to Future Climate

Common garden experiments are well suited for testing hypotheses regarding the consequences of assisted migration; however, our broader objective is to predict how natural populations will respond to future climate conditions. To visualize the projected climate shifts across the range of black spruce, we classified the climate state of each population in the past and across four future periods (2021–2040, 2041–2060, 2061–2080, and 2081–2100) under three carbon emission scenarios into 10 clusters (Figure S26). The West, Central, and East genetic clusters were projected to experience progressively warmer climates over time and along the south–north gradient (Figure S26).

Given the differences in model performance observed during validation, particularly between western and central/eastern common gardens, when depending on climate predictors, we adopted a region‐specific modeling approach. In the western part of the species range, genomic offsets were projected to increase across most of the region, reaching their highest values in the northwest (Figure 7A). In contrast, in the central and eastern regions, projected genomic offsets were generally higher in central populations than in eastern ones (Figure 7B). Given the variable model performance across gardens and genetic clusters, these projections should be regarded as exploratory.

**FIGURE 7:**
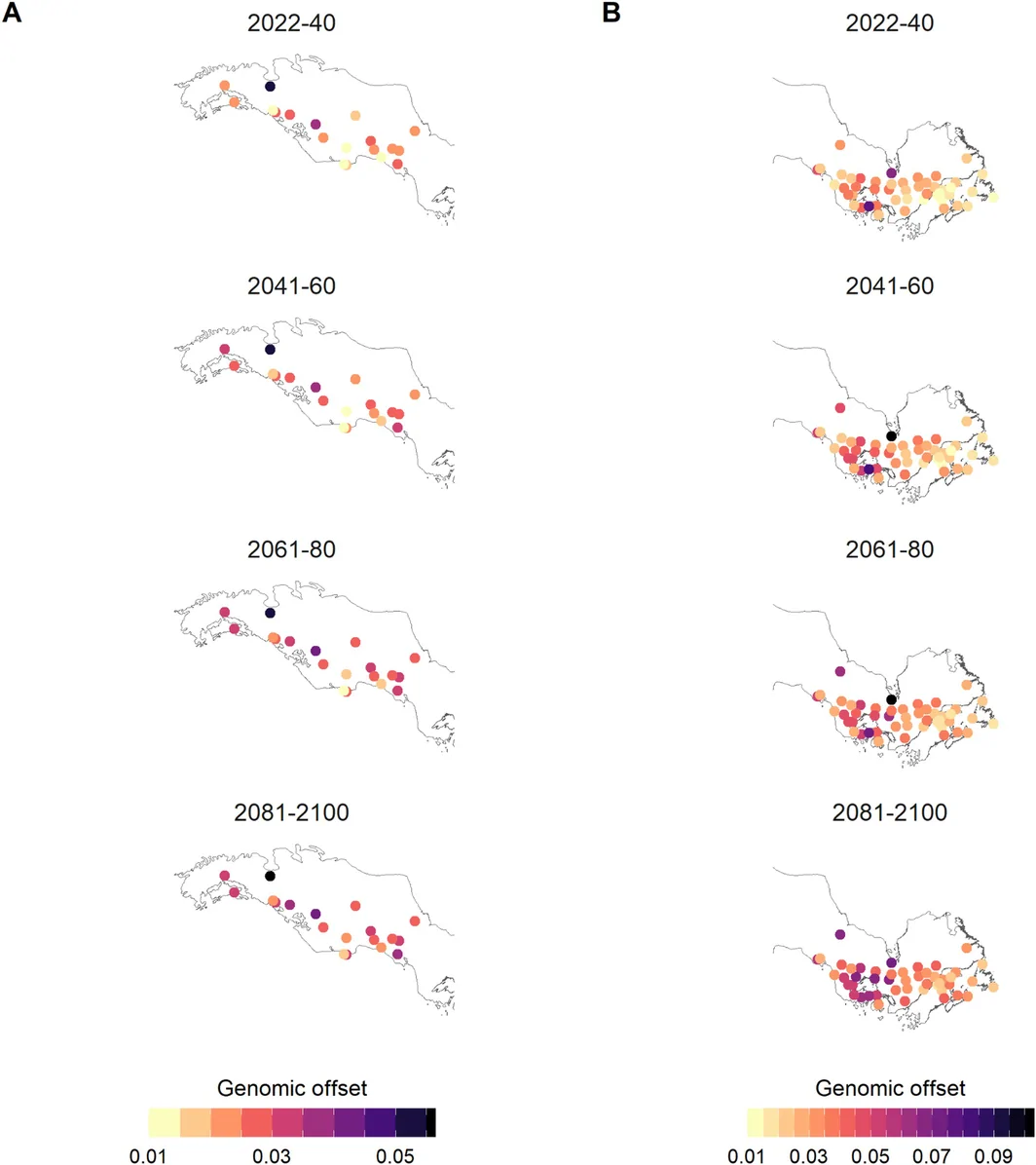
Projected genomic offsets under future climatic scenarios. Genomic offsets were projected for four 20‐year periods (2021–2040, 2041–2060, 2061–2080 and 2081–2100) under the medium emission scenario (SSP2‐4.5). They were projected separately for each region using the Gradient Forest model and climate predictors that performed best in validation for that region. (A) Genomic offset projected across four time periods using a model trained on the West cluster dataset and three climate variables (dPP, CMI and fallMT). (B) Genomic offsets projected across four time periods using a model trained on the combined dataset and three climate PCs. Genomic offsets indicate relative differences among populations within each region and should not be compared between panels A and B.

## 4 Discussion

### 4.1 Distribution of Genetic Diversity in Black Spruce

Black spruce carries high levels of genetic variation and has large estimated population sizes across its distribution due to its outcrossing mating system and wind pollination (Isabel et al. 1995; Perry and Bousquet 2001; Bouillé and Bousquet 2005). Our results confirm that this variation is sorted among three widespread genetic clusters: West, Central, and East, with the West cluster being the most distinct (Jaramillo‐Correa et al. 2004; Gérardi et al. 2010; Prunier et al. 2012). This structure is interpreted as the legacy of the Last Glacial Maximum (Jaramillo‐Correa et al. 2004), although our data do not exclude the possibility that the Central and East clusters were derived from a single glacial refugium, and later diverged after migrating and adapting to distinct environmental conditions. We also identified two isolated subclusters, one within the Central cluster (WI) and one within the East cluster (ME). While the distribution of WI is most likely limited to a small area, for ME it's difficult to judge how widespread this subcluster is, as it is situated at the southern limits of our sampling range, but not at the limit of the species range. Pairwise *F*_ST_s show these two subclusters are genetically differentiated from other clusters, from each other, as well as from the red spruce, which suggests that they could be remnants of glacial microrefugia that failed to expand across the landscape. Such cryptic refugia were identified in other North American species (Shafer et al. 2010; Fernandez et al. 2021).

Heterozygosity estimates based on variant sites only are driven in structured populations by the amount of divergence between populations, which determines the number of population‐specific invariant sites remaining in the dataset (Schmidt et al. 2021). This appears to be the case in black spruce, as observed heterozygosity was lowest in Western populations when all sites were considered, but highest when only polymorphic sites per population were included. This would suggest that Western populations do not necessarily have smaller effective population sizes than Central or Eastern populations.

### 4.2 Limitations to Genomic Offset in Black Spruce

Overall, our validation framework revealed that within common gardens, genomic offset projections were largely robust to model choice, marker number, or size of the dataset used for model training. In line with accumulating literature, the performance of genomic offsets was context dependent (Fitzpatrick et al. 2025), varying among common gardens, fitness traits selected for validation, and climate predictors used in the models. In addition, genetic structure influenced model outcomes, as genomic offsets estimated at the species level were not always consistent with those calculated within individual genetic clusters.

### 4.3 Genomic Offset and Climate‐Distance Projections Are Garden‐Dependent

A key assumption underlying genomic offset metrics is that local populations are under stabilizing selection for optimal phenotypes (Gain et al. 2023). Using common garden experiments, we demonstrated that population responses to climatic conditions varied depending on the garden environment, the traits measured, and the genetic background of each population (genetic cluster to which population belonged). Model performances varied mostly between gardens, with the strongest results observed in AC, and the weakest in PR, where genomic offsets were either not correlated or positively associated with fitness traits. Two reasons may account for the discrepancies observed in PR. First, the presence of noncrossover genotype‐by‐environment interactions, where populations from the Central cluster grow on average better than those from the West cluster (Robert et al. 2024). West cluster populations could exhibit lower relative fitness than Central populations despite being locally adapted. Second, populations from the West cluster may respond to a different set of climatic drivers than those included in the species‐wide models. Our results provide support for both scenarios. Genomic offsets and fitness traits were partly decoupled at the species level (Figure S27), likely driven by genotype‐by‐environment interactions affecting fitness rankings among populations. Moreover, model performance improved when different sets of climate variables were used (Figures 5 and 6), suggesting that species‐wide models may not fully captured the environmental drivers of adaptation in the West cluster.

Apart from the lack of model consistency across gardens, we also observed that model performance varied depending on the genetic cluster in which genomic offset was tested. This pattern further suggest that adaptive processes may operate differently among regions and are not adequately captured by species‐wide models. Such regional‐dependence resonates with recent findings conducted in genetically structured maritime pine ( *Pinus pinaster* Ait.), which found mixed results of offset‐fitness correlations across multiple common gardens (Archambeau et al. 2025).

In contrast to previous validation studies (Fitzpatrick et al. 2021; Lachmuth et al. 2024), genomic offset models rarely outperform climate transfer distance, both at species and cluster level. In theory, genomic offset is expected to model fitness loss better than climate because climate predictors are weighted by their effects on association with genetic variation (Gain et al. 2023). However, simulations of markers adapted to known causal environmental gradients in common gardens showed that climate‐based distance can be an equally good predictor of fitness as Gradient Forest‐based genomic offsets, in particular when causal environments are orthogonal and nonlinear (Láruson et al. 2022). Moreover, in Douglas‐fir, models trained on climate variables identified as influential for fitness in common garden experiments proved more accurate than those trained on other variables (Lind et al. 2024). Together, these findings suggest that climate PCs used in our models capture key environmental gradients driving fitness variation in most common gardens.

### 4.4 Projections Are Trait‐Dependent

The selection of fitness traits clearly influenced model performance, as not all traits are good predictors of environmental change. Height, total accumulated biomass, and DBH in general showed the strongest negative correlations with climate distance and genomic offsets. These traits are commonly used as proxies for fitness in tree species, because they respond to climatic variation in common garden experiments and show moderate heritability (Housset et al. 2018; Fitzpatrick et al. 2021; Depardieu et al. 2021; Girardin et al. 2021; Robert et al. 2024). Interestingly, short‐term accumulated biomass showed contrasting patterns across the gardens depending on tree age. In the two warmest gardens (ML and AC), early growth intervals of biomass were better associated with climate distance and genomic offsets, whereas in the two coldest gardens (PR and CH), later growth intervals showed the strongest associations, both at the species and cluster levels. Low correlations between juvenile and mature traits have been previously observed in conifers (e.g., Depardieu et al. 2021). In terms of genomic offset, these results only partially align with recent findings in red spruce, where stronger associations were observed for juvenile‐stage traits, when compared to later developmental stages (Verrico et al. 2025). As red spruce gardens were located at lower latitudes than those of black spruce, this discrepancy may reflect differences in temperature or photoperiod effects on early vs. late‐stage response of trees. Although survival can be a useful metric for fitness (Lind et al. 2024; Archambeau et al. 2025), we found it responded weakly to climate, mostly in PR and AC. This may be explained by the absence of early‐stage mortality in our measurements, possibly leading to this weak signal.

Mean drought‐related metrics also varied across gardens and genetic clusters but were only weakly associated with climatic distance or genomic offsets. An independent dendroecological study conducted in the same trials found that trade‐offs between drought resistance (Rt) and post‐drought recovery (Rc) varied only weakly among provenances, while their expression depended largely on drought intensity at each garden (Robert 2026). This pattern is consistent with the weak associations observed between drought‐related metrics and genomic offset.

### 4.5 Factors Influencing Model Performance

We did not find clear effects of model choice or marker number on model performance. This generally agrees with previous studies validating genomic offsets in common gardens or through simulations (Fitzpatrick et al. 2021; Láruson et al. 2022; Lind et al. 2024; Lind and Lotterhos 2025a; Verrico et al. 2025), and likely reflects that a large proportion of segregating variation in black spruce is associated with environmental gradients as a consequence of the spatial distribution of genetic clusters. Despite broadly similar model performance across marker sets, genome‐wide and neutral markers did not always produce the same genomic offset rankings, in particular for LFMM markers and RDA models. LFMM outliers displayed lower heterozygosity compared to other markers, suggesting a higher proportion of locally fixed alleles, and making them unequally informative across the species range.

Increases in model performance with larger training or test population sizes are expected when populations are genetically and phenotypically diverse, due to reduced sampling error caused by measurement or other nongenetic effects. In black spruce, a large proportion of genetic and phenotypic variation is distributed within populations (Rossi and Bousquet 2014). Consistent with this expectation, smaller test population sizes indeed led to weaker offset‐trait correlations in the best performing models. This pattern aligns with simulation studies showing that genomic offsets based on population‐level fitness means outperform those based on individual‐level measurements (Lind and Lotterhos 2025a).

In contrast, reducing the size of the training dataset or the number of populations included in it did not have a uniform impact on model training. One possible explanation is that, given the high genetic diversity of black spruce, even a limited number of randomly sampled populations may capture enough genetic variation and its association with climate to train effective models. In some cases, we even observed model improvement when a smaller number of individuals or a lower number of populations were used for training. This pattern could potentially arise if traits were correlated with genomic offset at the species level but not at the genetic cluster level–specifically, if correlation strength varied among clusters, meaning that the subsampling or removal of specific trees could inadvertently enrich the dataset for groups with stronger correlations.

Model performance in some gardens was strongly influenced by the selection of climate variables used for training. In particular, the two sets of climate predictors yielded contrasting results in AC and PR, indicating that predictor selection can have a decisive impact on model performance. This likely reflects the regional differences in the climatic factors limiting black spruce growth (D'Orangeville et al. 2018). In eastern regions, where precipitation is high, low temperatures limit growth in the north, whereas in the south, higher temperatures can constrain growth through higher evapotranspiration (Girardin et al. 2016; D'Orangeville et al. 2018; Lesven et al. 2024). In the western and central regions, lower precipitation makes moisture availability a major determinant of growth (Price et al. 2013; Girardin et al. 2016, 2024; Lesven et al. 2024). Because genomic offset measures the magnitude of climatic change regardless of its direction and assumes that populations are adapted to their local climate, climatic changes expected to increase growth (e.g., warming in cold‐limited regions) and those expected to decrease it (e.g., warming in moisture‐limited regions) are both interpreted as maladaptation. Consequently, no single model was able to accurately predict population responses to climate across the four common gardens. This finding further highlights the importance of identifying the key environmental drivers underlying genomic offset estimation and selecting predictors that reflect the ecological processes shaping fitness variation.

Previous studies pointed to the importance of the causal environmental predictors in certain conditions in genomic offset performance (Láruson et al. 2022; Lind et al. 2024). However, these drivers are rarely known a priori, and Gradient Forest does not always reliably identify them. Our results support this view, indicating that uncertainty in predictor choice is a key limitation of genomic offset approaches. A pragmatic strategy is to combine ecological knowledge (e.g., known growth‐limiting factors), sensitivity analyses using alternative subsets of predictors, and dimension‐reduction methods such as PCA, which summarize large sets of correlated climate variables into a few most evident “synthetic” gradients within a region. While latter approaches can improve model robustness, they may also complicate biological interpretation.

Region‐specific models are often expected to improve predictions by better capturing local drivers of adaptation. However, in our study they did not outperform species‐wide models, despite the presence of a pronounced genetic structure across the range. This result is noteworthy because these genetic clusters correspond to distinct glacial lineages that likely persisted in separate refugia during the last glaciation and followed different evolutionary trajectories after postglacial recolonization (Prunier et al. 2012). Previous studies using the same populations have also shown that climate responses differ among regions, with populations from the West cluster often exhibiting distinct growth responses and sensitivities to climatic stressors compared with populations from the Central and East clusters (Robert et al. 2024, 2025). Together these findings suggest that regional models may capture some cluster‐specific adaptive signals, whereas species‐wide models capture broader adaptive gradients shared across the range. In line with this interpretation, genomic offsets derived from models trained on different clusters were not always strongly correlated, suggesting that different training datasets can capture different components of adaptive variation. Nevertheless, these regional signals were not sufficient to reliably improve predictive performance relative to species‐wide models. Moreover, because future climates within a region increasingly resemble present‐day climates elsewhere in the species range, regional models are more likely to require extrapolation beyond their training range. The choice between regional and species‐wide models should therefore be guided by empirical validation performance and accompanied by an explicit assessment of climatic extrapolation.

## 5 Conclusions

By combining genome‐wide sequencing and phenotype data from distribution‐wide populations of black spruce grown in four long‐term common garden experiments, we evaluated the ability of genomic offset models to predict fitness in this genetically structured species. Although genomic offset was informative in some contexts, its predictive power was limited across the full species range. This limitation appears to arise, first, because similar ranks in genetic cluster relative fitness are retained between gardens, which prevents reliable projection of fitness across the species, and second, because species responses to climate vary across the landscape not only as a function of individual climatic variables but also by their interactions. Despite these limitations, models trained on carefully selected climate predictors proved to be robust not only to marker choice, but also to the number, size, and geographic range of populations used for model training. Future directions should prioritize the establishment of new common gardens, especially in western regions, where population responses to climate are less well understood. Improving genotype quality and increasing sampling density could also prove useful for refining genomic offset projections by enabling more accurate modeling of fine‐scale genotype‐environment associations. Finally, incorporating soil properties and associated microbiota in the future may help improve trait response curves under changing climate conditions.

## Funding

This study was funded by the Government of Canada through the Genomics Research and Development Initiative—Genomic Adaption and Resilience to Climate Change (GenARCC) project (2022–2027)—and by the Innovative Forestry Solutions and the Forest Climate Change programs of the Canadian Forest Service of Natural Resources Canada.

## Conflicts of Interest

The authors declare no conflicts of interest.

## Supporting information


**Dataset: S1** Tree sample information.
**Dataset: S2** Sampling coordinates of red spruce populations.
**Dataset: S3** Past climate normals for 43 climate variables.
**Dataset: S4** Population means and standard errors of measured phenotypic traits.

**Figure S1:** Patterns of isolation by distance across species distribution. Each point represents a pair of populations.
**Figure S2:** Four major axes of spatial autocorrelation. Each circle represents black spruce population. The two colors indicate either positive or negative values of the corresponding axes with size scaled by its absolute value.
**Figure S3:** Spatial visualization of sPCA four main axes. Each circle represents black spruce population. The two colors indicate either positive or negative values of the corresponding axes with size scaled by its absolute value.
**Figure S4:** Observed heterozygosity versus number of polymorphic sites across populations of black spruce. Observed heterozygosity was calculated from five random subsamples of seven individuals per population, after excluding monomorphic sites within each subsample. Colors correspond to geographical regions depicted in Figure 1A.
**Figure S5:** Performance of Gradient Forest (GF) and redundancy analysis (RDA) models validated against all fitness traits and estimated for all populations (“combined”) or individual genetic clusters. Triangles indicate Spearman rho calculated between trait value and climate transfer distance.
**Figure S6:** Comparison of predictive performance of Gradient Forest (GF) and redundancy analysis (RDA) models trained on eight different marker types and estimated for all populations. The marker types included subsets of 100 (0.1 K), 1000 (1 K) or all available markers (~29 K), subsets including low‐frequency variants (0.1 K‐lf, 1 K‐lf), and markers found to be in association with climate (LFMM, RDA, RDA‐struct).
**Figure S7:** Matrices of pairwise correlations between genomic offsets, estimated via Gradient Forest models trained on different sets of markers validated for the first eight fitness traits. The marker types included subsets of 100 (0.1 K), 1000 (1 K) or all available markers (~29 K), subsets including low‐frequency variants (0.1 K‐lf, 1 K‐lf), and markers found to be in association with climate (LFMM, RDA, RDA‐struct).
**Figure S8:** Matrices of pairwise correlations between genomic offsets, estimated via Gradient Forest models trained on different sets of markers validated for the last eight fitness traits. The marker types included subsets of 100 (0.1 K), 1000 (1 K) or all available markers (~29 K), subsets including low‐frequency variants (0.1 K‐lf, 1 K‐lf), and markers found to be in association with climate (LFMM, RDA, RDA‐struct).
**Figure S9:** Matrices of pairwise correlations between genomic offsets, estimated via RDA models trained on different sets of markers validated for the first eight fitness traits. The marker types included subsets of 100 (0.1 K), 1000 (1 K) or all available markers (~29 K), subsets including low‐frequency variants (0.1 K‐lf, 1 K‐lf), and markers found to be in association with climate (LFMM, RDA, RDA‐struct).
**Figure S10:** Matrices of pairwise correlations between genomic offsets, estimated via RDA models trained on different sets of markers validated for the last eight fitness traits. The marker types included subsets of 100 (0.1 K), 1000 (1 K) or all available markers (~29 K), subsets including low‐frequency variants (0.1 K‐lf, 1 K‐lf), and markers found to be in association with climate (LFMM, RDA, RDA‐struct).
**Figure S11:** Observed heterozygosity calculated per population using selected marker sets: all variants (All), LFMM outliers (LFMM), RDA outliers (RDA) and RDA outliers corrected for genetic structure (RDA‐struct). Minor allele frequency cutoff of 0.05 was applied to all datasets.
**Figure S12:** Comparison of predictive performance between models evaluated on training (axis *x*) and test datasets (axis *y*). Models were trained using Gradient Forest (GF) or redundancy analysis (RDA). Solid colors indicate model performances significantly different from zero. The marker types included subsets of 100 (0.1 K), 1000 (1 K) or all available markers (~29 K), subsets including low‐frequency variants (0.1 K‐lf, 1 K‐lf), and markers found to be in association with climate (LFMM, RDA, RDA‐struct).
**Figure S13:** Testing the impact of the number of populations in the training dataset on model performance. Points indicate five random draws of n populations (axis *x*). Lines connect the mean values for each population number. Gradient Forest (GF) and redundancy analysis (RDA) models were trained on a random set of 1000 markers.
**Figure S14:** Testing the impact of the population size in the training dataset on model performance. Points indicate 10 random draws of n genotypes (axis *x*). Lines connect the mean values for each population size. Gradient Forest (GF) and redundancy analysis (RDA) models were trained on a random set of 1000 markers.
**Figure S15:** Testing the impact of the population size in the test dataset on model performance. Points indicate 10 random draws of individual trees of a given number per population (axis *x*). Lines connect the mean values for each population size. Gradient Forest (GF) and redundancy analysis (RDA) models were trained on a random set of 1000 markers.
**Figure S16:** Gradient Forest model performances when trained on individual genetic clusters or combined datasets and three climate PCs in predicting fitness traits of the same or other genetic clusters. Dots indicate correlations with confidence intervals not overlapping zero. Models were trained on a random set of 1000 markers.
**Figure S17:** RDA model performances when trained on individual genetic clusters or combined datasets and three climate PCs in predicting fitness traits of the same or other genetic clusters. Dots indicate correlations with confidence intervals not overlapping zero. Models were trained on a random set of 1000 markers and three climate PCs.
**Figure S18:** Matrices of pairwise correlations between genomic offsets estimated using Gradient Forest models trained on single genetic clusters or combined datasets and three climate PCs. Models were trained on a random set of 1000 markers.
**Figure S19:** Matrices of pairwise correlations between genomic offsets estimated using RDA models trained on single genetic clusters or combined datasets and three climate PCs. Models were trained on a random set of 1000 markers.
**Figure S20:** Gradient Forest model performances when trained on individual genetic clusters or combined datasets and three selected climate variables in predicting fitness traits of the same or other genetic clusters. Dots indicate correlations with confidence intervals not overlapping zero. Models were trained on a random set of 1000 markers.
**Figure S21:** RDA model performances when trained on individual genetic clusters or combined datasets and three selected climate variables in predicting fitness traits of the same or other genetic clusters. Dots indicate correlations with confidence intervals not overlapping zero. Models were trained on a random set of 1000 markers.
**Figure S22:** Matrices of pairwise correlations between genomic offsets estimated using Gradient Forest models trained on single genetic clusters or combined datasets and three selected climate variables. Models were trained on a random set of 1000 markers.
**Figure S23:** Matrices of pairwise correlations between genomic offsets estimated using RDA models trained on single genetic clusters or combined datasets and three selected climate variables. Models were trained on a random set of 1000 markers.
**Figure S24:** Matrices of pairwise correlations between genomic offsets estimated using Gradient Forest models trained on two different climate datasets: a selected subset of three climate variables (“var3,” axis *x*) and three climate PCs (“PC3,” axis *y*). In this case models were trained on either complete datasets (“combined”) or individual genetic clusters. Models were trained on a random set of 1000 markers.
**Figure S25:** Matrices of pairwise correlations between genomic offsets estimated using RDA models trained on two different climate datasets: a selected subset of three climate variables (“var3,” axis *x*) and three climate PCs (“PC3,” axis *y*). In this case models were trained on either complete datasets (“combined”) or individual genetic clusters. Models were trained on a random set of 1000 markers.
**Figure S26:** Change of climate across the species range in the current century. A. Ordination of all population climate states (current and future projections according to three emission scenarios) along the first two principal components derived from 15 climate variables (arrows). On the plot climate variables are grouped by the type of variable, including variables linked to precipitation, variables describing low (Cold temp.) or high (Warm temp.) temperature regimes, mean annual temperature (MAT), minimum temperature in May (mayMinT) and photoperiod (dPP). B. Identified climate clusters present in the recent past (1961–1990) mapped onto species distribution. C. Predicted distribution of climate clusters in the four 20‐year periods (columns) between 2021 and 2100 according to three emission scenarios (rows).
**Figure S27:** Example of a confounding effect of differences in mean biomass increment between clusters on genomic offset estimates. Colored lines show linear models fitted separately to each cluster; dashed lines show linear models fitted to all samples. GF stands for Gradient Forest model and RDA for redundancy analysis prediction model that were used for calculating genomic offsets.
**Table S1:** Geographical location of all initial black spruce populations (provenances) and the number of retained tree genotypes in each common garden after filtering.
**Table S2:** Geographical location and mean climate conditions of common gardens.
**Table S3:** List of climate variables.
**Table S4:** Number of populations assigned to TEST and training (TRAIN) datasets. Datasets with less than 5 trees per populations were excluded from downstream analyses.
**Table S5:** Number of genotypes assigned to TEST and training (TRAIN) datasets.
**Table S6:** Results of AMOVA.
**Table S7:** Results of partial RDA analysis. Different classes of variables include genetic structure (gen), climate variables (clim) and spatial autocorrelation axes (spa).
**Table S8:** Partition of variance explained by climate in partial RDA models fitted to species‐wide or within‐cluster data. Models were run using climate as the only predictor (climate‐only) or while controlling for spatial (MEM) and genetic variables (pure climate). Adj stands for adjusted. Negative adjusted *R* ^2^ values were set to 0.

## Data Availability

Raw and filtered variant datasets are publicly available on Zenodo at https://doi.org/10.5281/zenodo.19961099. Datasets S1–S4 are archived at https://doi.org/10.5281/zenodo.20563726, and the associated analysis code is available at https://doi.org/10.5281/zenodo.20563774.
